# The Merits of a Two-Day Evidence-Based Medicine Course for Surgical Residents

**DOI:** 10.1007/s00268-016-3495-0

**Published:** 2016-03-10

**Authors:** Dirk T. Ubbink, Dink A. Legemate, Mark J. Koelemay

**Affiliations:** Department of Surgery, G4-184, Academic Medical Center at the University of Amsterdam, 1105AZ Amsterdam, The Netherlands

## Abstract

**Background:**

Over 10 years ago, we introduced a two-day, evidence-based surgery course for surgical residents. During the last 4 years, we evaluated its effect on the participants’ evidence-based medicine (EBM) knowledge and skills.

**Methods:**

Between 2012 and 2015, six courses were organised for residents of various surgical specialties of allied hospitals in the Amsterdam educational district. The courses covered the literature search, critical appraisal of surgical papers, and how to communicate and weigh the benefits and harms of surgical interventions. Proficiency regarding interpreting evidence was tested before and directly after the course using a modified Berlin questionnaire.

**Results:**

One hundred participants attended the courses, comprising residents in surgery (61 %), orthopaedics (16 %), urology (7 %), plastic surgery (7 %), and surgical PhD students (9 %), most of whom had already been taught EBM during their medical curriculum. Pre-course score levels were already fairly high (6.19 out of 10), but scores after the course were significantly higher (7.04); mean difference 0.85 (95 % confidence interval 0.4–1.3). No significant differences were observed among the surgical specialties. Attendees highly appreciated the course.

**Conclusions:**

A two-day, evidence-based surgery course improved EBM aptitude of surgical residents. Hence, the course appears useful to refresh the EBM paradigm among future Dutch surgeons.

## Introduction

Evidence-based medicine (EBM) was introduced in the early 1990s [1], coined by Guyatt and Sackett at the McMaster University in Hamilton, Canada. It has been defined as “the conscientious, explicit, and judicious use of current best evidence, in combination with the physician’s clinical expertise, patient preferences, and likely actions, in making decisions about the care of individual patients” [2].

Not long after the paradigm crossed the Atlantic to spread throughout Europe, it was embraced by general surgeons and orthopaedic surgeons in the Netherlands [3, 4]. The need for EBM was felt particularly in surgery, because evidence-based surgery (EBS) was lagging behind other medical realms like internal medicine, where pharmacotherapeutical research is easier to obtain than high-quality surgical research [5].

One of the initiatives to improve evidence-based thinking and practice in the Academic Medical Center in Amsterdam was the introduction of an evidence-based surgery course in 1999, which was open to staff members, residents, and PhD students of the major surgical specialties. In 2004, the Dutch Society for Surgery introduced a similar EBS course in its surgical training curriculum. A few years later, EBM was introduced as a standard topic in the curriculum of medical students. However, the present generation of clinicians has not (yet) received any formal EBM training. This tends to thwart the expectations of their fresh colleagues who are keen to apply their recently acquired EBM skills in clinical practice.

Previous studies have indicated that short courses in EBM are effective to enhance the knowledge of postgraduates [6], although skills and attitude are improved more if it is integrated in clinical practice [7, 8]. Hence, we investigated the effectiveness in terms of EBM proficiency and satisfaction with an interactive, two-day, EBS course for surgical residents in the educational region of the Academic Medical Center at the University of Amsterdam.

## Methods

Dutch surgical residents must attend this two-day evidence-based surgery course as part of their compulsory theoretical education during the first years of their surgical training. Participants can register via the website http://www.evidence-based-surgery.net. The course aims to teach the participants the principles and five steps of EBM, formulate concise clinical questions (using the PICO-structure), how to find the relevant literature, interpret the validity of the research, appreciate the results without the need for (knowledge about) statistical analyses, and apply these results to clinical practice and communicate these with their patients. We collected and analysed the results of the last six courses organised between 2012 and 2015.

### Course content

The course comes with a course manual, which contains a general introduction about EBM, introductory chapters on how to critically appraise various study designs based on relevant publications in the surgical literature, four surgical papers the participants are to appraise during the course, and a glossary of epidemiological terms. These papers were chosen on the basis of surgical relevance and didactic value and are regularly updated. For example, the study designs chosen and discussed in the most recent course were an observational study [9], a diagnostic accuracy study [10], a systematic review [11], and a randomised clinical trial [12].

The manual also provides specific checklists, based on the Dutch Cochrane Centre (http://www.dcc.cochrane.org/) and the *Users’ guides to the medical literature* produced by the EBM Working Group from McMaster University, Canada [13], to guide participants when critically appraising each of the study designs. Participants receive the manual well in advance and are strongly advised to read it in order to get the most out of the course.

The first day of the course starts with a the presentation on the five steps of EBM and a clinical case presented during the morning handover, which could have happened the night before and the participants may have had to deal with: A 60-year-old healthy male who tripped and fell on his right wrist. The X-rays are presented showing a comminuted intra-articular distal radius fracture. The participants engage in a discussion about how they would treat this wrist fracture and what the evidence behind their choice is. Then they attend a workshop, organised by one of the clinical librarians, to help them find the relevant literature in general, using PubMed and the Cochrane Library, and in particular about this clinical case. Next, a presentation is given about the value and how to interpret an observational study, which is still the most common study design in surgery. This is followed by small-group workshops (about 8 participants each) in which they critically appraise such a study, coached by a mentor. Lastly, a presentation is given on the value of diagnostic tools in surgery and how to interpret a diagnostic accuracy study, again followed by a workshop.

The second day of the course comprises a presentation on the value and interpretation of systematic reviews and meta-analyses, followed by a workshop. In another presentation, the benefit and harm of surgical interventions are discussed and how to weigh these in clinical decision making [14]. This is also discussed in a workshop. The course is wrapped up by letting the participants give feedback on the evidence they found about the clinical case, and inviting them to give an overall evaluation of the course.

All speakers and workshop mentors are surgeons and/or clinical epidemiologists, affiliated to the Department of Surgery and with ample experience in teaching and practicing EBM.

### Effectiveness measurements

Assessment of the baseline and post-course EBM level among the participating residents was based on the Berlin questionnaire [6], which evaluates individual knowledge about interpreting evidence. This was considered the best test to appreciate the critical appraisal of study designs and interpretation of study results as trained during the course, and has been used for this purpose before in various settings [15, 16]. The questionnaire has been translated and validated in Dutch [17].

The questions for this course were slightly modified in that they were rephrased using surgical scenarios and again adapted for the post-course exam. The two sets were applied randomly for the pre- and post-course tests. Examples on the interpretation of study results and critical appraisal of study designs are shown in Table 1. Also, the original questionnaire was shortened from 15 to 10 multiple-choice questions. These questions were to be answered within 15 min.

**Table 1:** Examples of two questions from the Berlin questionnaire adapted for surgical residents

1. A large double-blinded RCT showed that preoperative statin therapy reduced the risk of a lethal perioperative myocardial infarction by 50 %. In the experimental group 4 out of 4000 (0.1 %) patients died, in the placebo group 8/4000 (0.2 %)
How many patients should be treated with a statin to prevent one additional death due to a myocardial infarction?
A. 1000 = (1/ (0.2–0.1 %)
B. 2000 = (8000/4)
C. 4000 = (4 × (1/0.1 %))
D. 8000 = (4000 × 2)
E. Can’t tell based on these data
2. Which statement about meta-analyses is true?
A. Larger sized studies produce a larger treatment effect
B. It suffices to include English publications in a meta-analysis
C. Due to meta-analyses the need for large RCTs has diminished
D. Differences in primary studies (e.g. population of research question) can be corrected by means of statistical techniques
E. None of these statements is true

Satisfaction with the content, presentation and organisation of the course was measured using 20 items (as shown in Table 2) to be answered on a semi-quantitative scale, ranging from ‘bad’ to ‘excellent’. The scores were expressed on a scale from 0 to 10, including their standard deviations.

**Table 2:** Evaluation questionnaire applied in EBS courses

### Data analysis

The results of the Berlin test before and after the course were expressed as mean values, after testing for a normal distribution. These values were compared by calculating the mean difference with its 95 % confidence interval (CI). A possible influence of the specialty of the residents and the year in which the course was given was investigated using analysis of variance (one-way ANOVA).

The results of the satisfaction questionnaire were expressed as mean values, including their ranges at course level.

## Results

During the 3.5 year period, a total of 100 participants attended the course. These were residents in training to become a (gastrointestinal, vascular, paediatric, trauma, or neuro-) surgeon (61 %), orthopaedic surgeon (16 %), urologist (7 %), plastic surgeon (7 %), and clinical Ph.D students (9 %). Some of them, and if so, especially the PhD students, had received prior training in clinical epidemiology or EBM. All participants completed the questionnaires. Two out of the 100 did not complete the initial, and 9 did not complete the final assessment.

Participants rated the overall quality of the course with 8.1 out of 10 (range per course: 7.8–8.5). In particular, they highly appreciated the content, form, and organisation of the course (Table 3), mainly because of its strong focus on clinical surgical practice. The clinical scenario at the start of the course usually confronted the participants with their uncertainty about the best treatment option and their limited knowledge about the existing evidence to support this. However, during the course the participants experienced that the training how to search and critically appraise available evidence had empowered them, or had refreshed their ability, to apply EBM in clinical practice in their own hospitals. Many participants felt strengthened to introduce or promote this paradigm in their own hospitals and to challenge their supervisors regarding the evidence behind their treatment choices.

**Table 3:** Results of course evaluation

Item	Score (range)
Overall	
1. Course content	8.0 (7.5–8.5)
2. Play a role	8.9 (8.7–9.2)
3. Express opinion	8.8 (8.3–9.2)
4. Course manual	7.6 (6.8–8.0)
5. Course organisation	8.4 (8.0–8.8)
6. Course location	7.5 (7.0–8.0)
7. Catering	9.2 (8.8–9.5)
8. Overall quality	8.1 (7.8–8.5)
Presentations	
9. Clinical problem	
P	8.0 (7.8–8.2)
C	7.8 (7.6–8.1)
10. Introduction	
P	8.0 (7.7–8.2)
C	7.8 (7.5–8.0)
11. Observational studies	
P	7.5 (6.5–8.0)
C	7.5 (6.0–8.0)
12. Literature search	
P	6.0 (4.5–6.8)
C	6.3 (5.0–7.0)
13. Diagnostic accuracy studies	
P	7.8 (7.3–8.0)
C	7.9 (7.5–8.3)
14. Systematic reviews	
P	7.7 (6.8–8.0)
C	7.6 (7.0–8.0)
15. Benefit versus harm	
P	8.0 (7.5–8.5)
C	7.8 (7.3–8.3)
16. Feedback on literature search	
P	6.8 (6.0–7.8)
C	6.8 (6.0–8.0)
Workshops	
17. Observational study	8.2 (7.8–8.8)
18. Diagnostic accuracy study	8.3 (7.5–8.8)
19. Systematic review	8.2 (7.5–8.8)
20. Benefit versus harm	8.0 (6.8–6.5)

Scores are presented on a 10-point scale; 0 is the lowest, 10 is the highest score with their ranges (at course level)

*P* presentation; *C* content

The mean scores of the modified Berlin questionnaire increased from a pre-course value of 6.2 out of 10 (SD 1.7) to a post-score value of 7.1 out of 10 (standard deviation (SD) 1.3). This increase was statistically significant: mean difference 0.85 (95 % CI 0.46–1.25), with an effect size (difference in means divided by the SD) of 0.57, which is moderate to large [8]. Although we found a significantly higher (*P* = 0.017) mean increase in the 2013 cohort (difference = 2.3) than in the other cohorts (difference = 0.61), we did not observe a trend during the years the course was given or between the distinct courses, nor could we detect any statistically significant difference (*P* = 0.78) among the residents’ specialties.

## Discussion

The two-day, interactive evidence-based surgery course for surgical residents was found to improve EBM aptitude and willingness to apply in daily clinical practice.

The increase in Berlin scores was slightly less than the study by Fritsche et al. [6] (showing a rise from 3.9 to 6.3 out of 10). Probably, this is due to the fact that the entrance EBM level of most of the participants was relatively high. Nevertheless, their scores further improved directly after the course, demonstrating that even then the course has a beneficial effect on the participants’ knowledge to interpret surgical research. This was corroborated by our effect size, which was slightly larger than in a previous study [16]. Although not quantified, the introduction of a compulsory EBM course for surgical residents in the Netherlands has led to more integration of EBM features (e.g. the formulation of PICOs and the production of critically appraised topics; CATs) in within- and between-hospital surgical research meetings and grand rounds.

Not all EBM skills (e.g. formulation of the clinical question, search competency, application to the patient, and EBM attitude) taught in the course were captured by the questionnaire. As an alternative to the Berlin questionnaire, the Fresno test could have been used [18]. Both evaluate all four steps of EBM [19]. The Fresno test requires participants to perform realistic EBM tasks, demonstrating applied knowledge and skills. However, more time and expertise are required to grade this instrument. The multiple-choice format of the Berlin questionnaire not only assesses EBM-applied knowledge but also makes it more feasible to implement. The ultimate, long-term aim of the course, improved application of EBM in clinical practice, was not investigated in this study. For this purpose, other instruments are available [20].

According to its definition, EBM also includes incorporation of the patients’ preference as to the possible treatment options, besides the integration of best available evidence in deciding about a treatment choice [1]. Apparently, the focus on collecting and appreciating high-quality evidence for clinical practice has downplayed the importance of risk communication and the role of the patient in treatment decision making [21]. These aspects are gradually receiving more attention in current EBM courses for clinicians and medical students.

Finally, we realise that this course should be evaluated in other settings and countries to further appreciate its merits. We hope that this publication will foster its dissemination in order to reach this goal.
